# Exploiting explanations for model extraction via knowledge distillation and mitigation with private counterfactuals

**DOI:** 10.3389/frai.2026.1746910

**Published:** 2026-05-25

**Authors:** Fatima Ezzeddine, Silvia Giordano, Omran Ayoub

**Affiliations:** 1 Università della Svizzera italiana, Lugano, Switzerland; 2 Scuola universitaria professionale della Svizzera italiana, Lugano, Switzerland

**Keywords:** differential privacy, explainable AI, knowledge distillation, model extraction attacks, responsible AI deployment

## Abstract

In recent years, there has been a notable increase in the deployment of machine learning (ML) models as services (MLaaS) across diverse production software applications. In parallel, explainable AI (XAI) continues to evolve, addressing the necessity for transparency in ML models. XAI techniques aim to enhance the transparency of ML models by providing insights, in terms of *model's explanations*, into their decision-making process. At the same time, some MLaaS platforms now offer explanations alongside the ML prediction outputs. This setup has elevated concerns regarding vulnerabilities in MLaaS, particularly in relation to privacy leakage attacks such as model extraction attacks (MEA). This is due to the fact that explanations can unveil insights about the inner workings of the model which could be exploited by malicious users. In this work, we focus on investigating how model explanations, particularly counterfactual explanations (CFs), can be exploited for performing MEA within the MLaaS platform. We also delve into assessing the effectiveness of incorporating differential privacy (DP) as a mitigation strategy. To this end, we first propose a novel MEA approach based on Knowledge Distillation (KD) that leverages CFs to effectively extract a substitute model of the target. Our approach operates without requiring any prior knowledge of the training data distribution by the attacker. Then, we advise an approach for training CF generators integrating DP to generate private CFs. We conduct thorough experimental evaluations on real-world datasets and demonstrate that our proposed KD-based MEA can yield a high-fidelity substitute model with a reduced number of queries with respect to baseline approaches. Furthermore, our findings reveal that including a privacy layer can allow mitigating the MEA. However, by balancing the quality of CFs, impacts the performance of the explanations and the MEA.

## 1 Introduction

Recent years have witnessed a growing trend in employing deep neural networks (DNNs) as learning algorithms for machine learning-based applications. In particular, DNNs have gained substantial popularity due to their remarkable success in diverse domains (Shinde and Shah, 2018). However, the complexity of their training process is resource intensive, as it involves the acquisition of data and requires substantial computational power (LeCun et al., 2015), and therefore hinders their widespread adoption and accessibility. One approach to mitigating these complexity challenges is by offering machine learning (ML) models through dedicated ML as a Service (MLaaS) platforms (Nasr et al., 2019; Shokri et al., 2021; Aslani and Ghobaei-Arani, 2025). MLaaS platforms streamline the accessibility to complex models by hosting and training ML models on the cloud, allowing third-party practitioners to query pre-trained models through Application Programming Interfaces (APIs) (Wang et al., 2022; Aslani and Ghobaei-Arani, 2025), while the private dataset used for training the model remains inaccessible to users.

Numerous platforms offer MLaaS such as Google Cloud, Microsoft, IBM, and Amazon Web Services (Google Auto ML, 2024; Microsoft Explainable AI, 2024; Google Explainable AI, 2024; AWS Explainable AI, 2024). The API's input-output format for these services is publicly accessible, meaning that users have knowledge of the format of input data required by the service (by the ML model), and therefore can receive decisions from the model. This opens the door for malicious users (or, more precisely, attackers) to attempt to exploit these interfaces to conduct privacy-breaching attacks such as membership inference attacks (MIA) (Shokri et al., 2017), model inversion attacks (MINA) (Wang et al., 2021), and model extraction attacks (MEA) (Tramèr et al., 2016). For instance, in MIA, an attacker aims to infer the presence of a specific individual instance in the private training set. In MINA, the attacker attempts to reconstruct the training set. In MEA, the attacker attempts to extract the model by training a substitute model using data acquired through repetitive queries to the service provider's confidential model.

Several mitigation strategies for these security- and privacy-breaching attacks are already employed (Dwork et al., 2006; Ghosh et al., 2025). However, these strategies face new challenges as emerging transparency requirements demand explanations for ML model decisions. In response, MLaaS platforms are shifting toward providing explanations alongside the predictions made by their deployed models (Microsoft Explainable AI, 2024; Google Explainable AI, 2024; AWS Explainable AI, 2024). The need to provide users with model explanations is linked to providing users with a transparent decision-making process of data-driven systems (Nguyen et al., 2024). The urge to enhance transparency through model explanations stems from a dual objective: on the one hand, to satisfy emerging regulatory and compliance requirements, and on the other hand, to offer users actionable insights derived from the model's decision-making process (Juliussen, 2025; Panigutti et al., 2023; Nguyen et al., 2023; Thang et al., 2015; Nguyen et al., 2024).

Model explanations are generally derived using explainable AI (XAI) techniques, which are designed to offer insight into the reasoning process behind a model's decision (Holzinger et al., 2019; Ribeiro et al., 2016). However, these explanations may unintentionally reveal information that can be exploited by attackers to refine their strategies, thereby opening new opportunities for existing attack methodologies, and posing additional risks to current security and privacy safeguards (Huang et al., 2024, 2023; Pawelczyk et al., 2023).

In this work, we focus on investigating how explanations generated by XAI frameworks, such as counterfactual (CF) explanations, can be exploited to conduct MEA in MLaaS scenarios. We precisely focus on CFs due to the unique insights they provide. CFs reveal how minimal alterations to original data instances can lead to different model outcomes, consequently helping users interpret the dynamics underlying the model's prediction shift from the decision boundary (Došilović et al., 2018). However, due to their inherently proximity to the decision boundary and their reflection of the training data, CFs exhibit a dual nature: while they serve as valuable interpretative tools, but also as potential assets by revealing additional information that can be exploited to uncover model vulnerabilities. In this context, we first propose a novel approach based on knowledge distillation (KD) to perform high-fidelity MEA which leverages CFs as a surrogate training dataset. Our method derives a threat model that closely replicates the functionality of the target model being.^1^ Moreover, we propose a mitigation approach that integrates differential privacy (DP) within the CF generator training pipeline, aiming to reduce the risk associated with providing CF explanations. Our analysis evaluates the effectiveness of the proposed attack method in comparison to established baselines and the impact of DP on both the quality of CF and the performance of the MEA.

The contribution of our work can be summarized as follows:

- **KD-Based MEA with CFs**: we focus on leveraging CF through a novel method founded on KD to perform MEA in MLaaS with a specific focus on MEA facilitated by KD as extraction techniques and using CFs as informative proxies for the original training data. To substantiate the effectiveness of this method, we simulate an adversarial scenario in which an attacker employs the proposed KD-based method to train a high-fidelity substitute model of the target model.
- **Private counterfactual explanations**: acknowledging the critical importance of preserving the privacy of training data, we introduce the integration of DP into the GAN-generated CF explanation pipeline. This enhancement is designed to generate CFs that deviate from the statistical properties of the sensitive dataset, thereby offering a layer of protection against potential privacy breaches. We also investigate the tradeoff associated with the integration of DP on the generated CFs, assessing its impact on both privacy and utility.

## 2 Preliminaries

In this section, we discuss the key concepts used in our work, namely, CF explanations, KD and DP.

### 2.1 Counterfactual explanations (CF)

**Figure 1:**
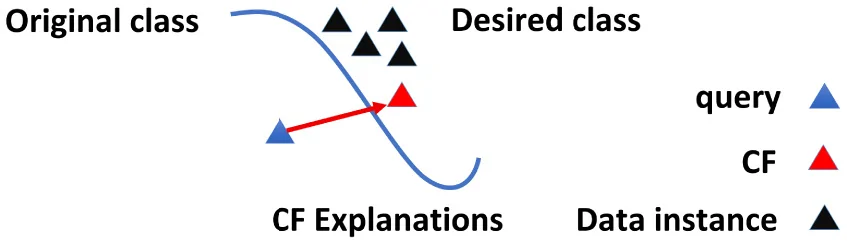
CF example involving navigating the decision boundary to find the optimal change in input for a desired outcome.

A CF explanation (Figure 1) is a form of example-based explanation that provides a hypothetical scenario that illustrates how a different decision or outcome of an underlying ML model could have been achieved through minimal changes to the input data (Wachter et al., 2017). CFs offer users an actionable explanation by showing how alterations to the input would influence the model's output (Poyiadzi et al., 2020). Additionally, CFs allow the identification of the variables that should have differed in a given input to observe a different outcome, thus making it possible to assess the influence of specific factors (Wachter et al., 2017). CF has already proven to improve the interpretability of ML models in several domains, such as healthcare and finance (Došilović et al., 2018; Cobzaru, 2025; Kumar et al., 2025).

To generate CFs, various CF explanation methods obtain CFs by optimizing a customized cost function. These explainers employ distinct strategies such as mixed-integer linear optimization (Kanamori et al., 2021), heuristic search strategies (Martens and Provost, 2014; Moore et al., 2019), and metaheuristic approaches (Sharma et al., 2019; Hashemi and Fathi, 2020; Numeroso and Bacciu, 2020; Nguyen et al., 2021), such as genetic algorithms (Sharma et al., 2019; Hashemi and Fathi, 2020), Reinforcement Learning (Ezzeddine et al., 2023; Chen et al., 2021), GANs (Nemirovsky et al., 2022) or Graph density bases (Poyiadzi et al., 2020). In this paper, we employ the method of Generating CFs for Real-Time Recourse and Interpretability using Residual GANs (CounteRGAN) as a CF generator (Nemirovsky et al., 2022). CounteRGAN is a novel residual GAN that trains the generator to produce residuals that are intuitive to the notion of perturbations used in CF searches. The search process seeks to maximize the value function concerning the discriminator *D* and minimize it concerning the generator *G*. Where *C*_*t*_ is the target classifier to be explained, Reg(G, *x*_*i*_) is a regularization term, and *x*_*i*_ are samples drawn from the entire data distribution (Nemirovsky et al., 2022) (Equation 1).


$$\begin{array}{l} V_{\text{CounteRGAN-bb}}\left(D,G\right)=\frac{\sum_{i}C_{t}\left(x_{i}\right)\log D\left(x_{i}\right)}{\sum_{i}C_{t}\left(x_{i}\right)}\\ \;+\frac{1}{N}\underset{i}{\sum }\log\left(1-D\left(x_{i}+G\left(x_{i}\right)\right)\right)\\ \;+\text{Reg}\left(G,\left\{x_{i}\right\}\right), \end{array}$$ (1)

CF generators aim to identify in-distribution data points from the training data points by optimizing metrics of proximity, plausibility, sparsity and proximity (Mothilal et al., 2020) (described later in Section 5.6). Specifically, *sparsity* measures how many features of the CF data point are different from the original data point while *proximity* measures the overall distance between the CF and the original data point, such as euclidean and gower distance, where a low proximity value indicates that the CF is similar to the original point. This can be useful for providing actionable feedback, as it suggests that the model is recommending only a few changes that are likely to have a significant impact on the output. *Plausibility* ensures that the generated CFs are realistic and fit in the training data distribution.

### 2.2 Knowledge distillation

Knowledge distillation (also known as model distillation), is a process that involves the transfer of knowledge from a high-complex model, known as a *teacher model*, to a reduced-complexity model, known as a *student model*, that remains operable within real-world constraints (Cho and Hariharan, 2019). While KD constitutes a specific instance of model compression, it serves as a means to extract essential insights, patterns, and expertise embedded in the larger models to create a deployable model. The student model follows a training procedure with the primary objective of imitating the performance of the teacher model. This knowledge transfer mechanism involves the student model learning not only the surface-level predictions made by the teacher model but also the deeper patterns, generalizations, and decision-making strategies embedded within the teacher architecture. To achieve this, the student model is guided during training by incorporating two primary sources of information: the actual target labels or predictions for the dataset at hand, and the soft labels or probability distributions generated by the teacher model in response to the same dataset. The rational for using KD for the MEA, is its unique capabilities to handle the problem not just as a standard classification task but as an estimation of the output probability distribution.

KD necessitates the availability of a well-trained teacher model, a trainable student model, and the specification of a student loss function for assessing predictions against ground-truth labels (*L*). A distillation loss function, accompanied by a temperature parameter (*temp*), is employed to bridge the knowledge gap by comparing the soft predictions of the student to the softened teacher labels. The student and the distillation losses are weighted via an alpha (α) which is essential for balancing task-specific accuracy and knowledge transfer. Subsequently, the losses incurred are computed, incorporating a weighted combination of the student loss (weighted by α) and the distillation loss (weighted by 1 − α). This weighting mechanism allows for a fine-tuned balance between preserving task-specific accuracy (student loss) and incorporating the knowledge distilled from the teacher model (distillation loss). The Kullback-Leibler (KL) divergence, denoted as *KL*(*P* ∥ *Q*) (Equation 2), is a mathematical measure employed in KD to assess the difference between two probability distributions, *P* and *Q*, where the summation is taken over all classes or categories.

Suppose *P* represents the soft probabilistic predictions of a teacher model, while *Q* represents the corresponding probabilistic predictions of a student model (expressed as the output made by a softmax activation function). During training, KL divergence as the distillation loss encourages the student model to capture the nuances and uncertainties present in the teacher's predictions. In summary, the loss to be optimized by the student is shown by Equation 3.


$$\begin{array}{l} distillation\text{\_}loss=KL\left(P∥Q\right)=\underset{i}{\sum }P\left(i\right)\log\left(\frac{P\left(i\right)}{Q\left(i\right)}\right) \end{array}$$ (2)

$$\begin{array}{l} loss=\alpha \cdot student\text{\_}loss+\left(1-\alpha \right)\cdot distillation\text{\_}loss \end{array}$$ (3)

### 2.3 Differential privacy

Differential privacy is a mathematical framework applied to safeguard individual records by introducing controlled noise to the data and allowing the extraction of valuable insights while ensuring that individual identities remain protected (Dwork et al., 2006). The introduction of noise to data allows analyzing data without disclosing sensitive information about any specific individual in the dataset (Dwork, 2006). DP aims to guarantee, by definition, that the inclusion or exclusion of any individual record in the dataset should have minimal impact on the outcome of the mechanism, where a mechanism refers to any mathematical computation applicable to and with the data. In the context of classification tasks, for instance, DP analyzes how the output undergoes probabilistic changes based on a given input. Hence, a mechanism guarantees DP if the likelihood of any outcome is nearly identical for any two datasets that vary by only one record.

One widely utilized approach for addressing numerical inquiries involves the incorporation of randomized approaches that add calibrated random noise, which works by introducing sufficient noise to the input or output of the mechanism to obscure the potential contribution of any individual record in the data while simultaneously maintaining the overall accuracy of the analysis. In Def. 1, we detail the DP inequality.

**Definition 1** (Differential Privacy). *A randomized algorithm* *M* *with domain* ℕ^2^ *is* (ε, δ)-*differentially private if for all* *S* ⊆ *Range*(*M*) *and for all* *x, y* ∈ ℕ^2^ *such that* ||*x* − *y*||_1_ ≤ 1:


$$\mathrm{Pr}\left[M\left(x\right)=S\right]\leq e^{\varepsilon }\cdot \mathrm{Pr}\left[M\left(y\right)\in S\right]+\delta ,$$

Def. 1 states that an algorithm *M* (ε, δ) is differentially private if, for all subsets *S* of the range of *M* and all pairs of inputs *x* and *y* differing by at most one data instance, the probability of *M*(*x*) outputting *S* is bounded by a factor of *e*^ε^ and an error margin δ.

In addition to the perturbation of input or output data, it is possible to achieve DP during the training of ML models and safeguard the privacy of the training model itself. This is usually achieved by introducing perturbations to the model weights and gradients during training (Abadi et al., 2016; Zhang et al., 2012). The process proceeds as follows: consider an ML model, to be trained on dataset *D*, with a parameter set w^*^ will minimize an objective $L_{D}\left(w\right)=\underset{t_{i}\in D}{\sum }L\left(t_{i},w\right)$, DP is injected during the optimization process to prevent the model from memorizing specific details about individual data points. DP training involves multiple steps of adding noise to the gradient of the model parameters concerning the training data being trained on, followed by gradient clipping. Then, the resulting parameter set w^*^, minimizes the loss that can be later derived.

## 3 Related work

Several works have proposed strategies for performing MEA. Authors in Tramèr et al. (2016) conduct successful MEA on different ML models such as decision trees, Support Vector Machines, and DNNs by using equation-solving and path-finding algorithms and learning theory. In Pal et al. (2020), authors conduct MEA on DNNs using active learning with unannotated public data. In Krishna et al. (2020), authors perform MEA in natural language processing on bert-based APIs, in which they explore leveraging transfer learning. Other works have focused on proposing querying strategies for MLaaS to effectively query the target model and extract accurate insights in addition to conducting MEA (Orekondy et al., 2019; Juuti et al., 2019; Oh et al., 2019). In Orekondy et al. (2019), authors train a knockoff network with queried image predictions and propose a reinforcement learning-based approach that enhances query sample efficiency and provides performance gains. In Juuti et al. (2019), the authors first propose a new method that generates synthetic queries and optimizes training hyperparameters, and then propose a method to detect effective DNN MEA. Other works focused on introducing a watermarking mechanism designed for text generated by Large Language Models (LLMs) to address copyright concerns and track content origin (Pang et al., 2025). Meanwhile, Zhang et al. (2025) proposes a new metric to quantify the risk of MEA by measuring the distance of weight changes between the target and its surrogate. Authors in Sun et al. (2025) explore existing obfuscation methods and propose a highly efficient new model obfuscation technique to protect against such attacks. In Oh et al. (2019), authors investigate the type and amount of internal information about the deployed ML model that can be extracted, such as the architecture, optimization procedure, and training data. Authors in Jagielski et al. (2020) enhance the query efficiency of MEA attacks by introducing learning-based methods and focusing on the fidelity of the extracted model.

Other works have investigated MEA of ML models using explanations provided by the MLaaS (Aïvodji et al., 2020; Oksuz et al., 2023; Yan et al., 2022; Sokol and Flach, 2019; Milli et al., 2019). In Oksuz et al. (2023), authors investigate how Local Interpretable Model-agnostic Explanations (LIME), an XAI framework, can be exploited to infer the decision boundaries by sending adaptive queries to generate new data samples that are close to the decision boundaries. In Miura et al. (2024), authors analyze how gradient-based explanations reveal the decision boundary of a target model by modeling a data-free MEA against a gradient-based XAI, in addition to exploiting a generative model to reduce the number of queries. Yan et al. (2022) proposed a methodology to conduct MEA by minimizing task-classification loss and task-explanation loss. Similar to our work, other works have investigated how CFs can be exploited for conducting MEA. In Aïvodji et al. (2020), authors model an attack that relies on both the predictions and the CFs of the target model to directly train an attack model. The authors in Wang et al. (2022) model a strategy to reveal the decision boundary of a target model and then perform MEA by considering the CF of the CF as pairs of training samples to directly train the extracted model.

Similar to these works, our work aligns with exploring how CF explanations can be exploited for performing MEA. However, our contribution lies in modeling a novel approach to MEAs that exploits more effectively the insights that CFs carry with respect to other approaches that directly train a substitute model on extracted CFs. Moreover, in contrast to previous studies (Aïvodji et al., 2020; Wang et al., 2022), our work presumes that the attacker possesses no prior knowledge of the training set, leading them to query the model with zero knowledge, and proposes a novel approach for performing the MEA.

In terms of mitigation strategies, few works have focused on proposing methodologies to generate explanations while mitigating revealing sensitive insights about the decision boundary, the training set, or the model architectures. For instance, authors in Patel et al. (2022) propose employing DP algorithms to construct feature-based model explanations, while authors in Yang et al. (2022) propose an approach to generate differentially private CFs via functional mechanisms. Pentyala et al. (2023) proposes an approach for generating recourse paths leveraging differentially private clustering and demonstrates that constructing a graph on the cluster centers provides private recourse paths as CFs.

Our work also exploits DP, however, by integrating DP into the CF generation process. By this approach, we eliminate the necessity for a distinct method to generate private CFs. We specifically focus on GANs-based CF generators due to their ability to generate high-quality CFs compared to other CF generation methods. To the best of our knowledge, our work is the first to integrate DP within CF generative-based methods. We show the robustness of the DP-based proposed method against MEA, and we provide a detailed analysis that sheds light on the impact of CFs in exposing the distribution of the training set through well-defined metrics.

## 4 Problem formulation and methodology

A DNN model *f*_θ_ trained on a dataset D: $X\in {\mathbb{R}}^{d}\to Y\in \mathbb{R}$ takes a numerical input query x∈X and predicts the output y∈Y. *f*_θ_ a trained model with θ be the weight matrix, *b* be the bias vector. The output of the DNN can be computed as *f*_θ_ = θ · *X* + *b*. The output of the network *y* are confidence scores, which are expressed in terms of probabilities, essentially indicating how confident the ML model is about each possible class or category in its training data. The CF explanation method, which trains a CounteRGAN generator *E*: $f_{\theta }\times X\to X$ generates a perturbed instance c∈X for the input instance *x* such that *f*_θ_(*c*) has a different output while minimizing an objective function *g*. Formally, searching for a CF can be framed as *argmin* *g*(*x, c*) *s*.*t* *f*_θ_(*c*) ≠ *f*_θ_(*x*). Where $g:X\times X\to R_{+}$ is a cost metric measuring the changes between the input *x* and *c*, and *y* is the desirable target that is different from the original prediction *f*_θ_ as shown in Figure 1.

**Figure 2:**
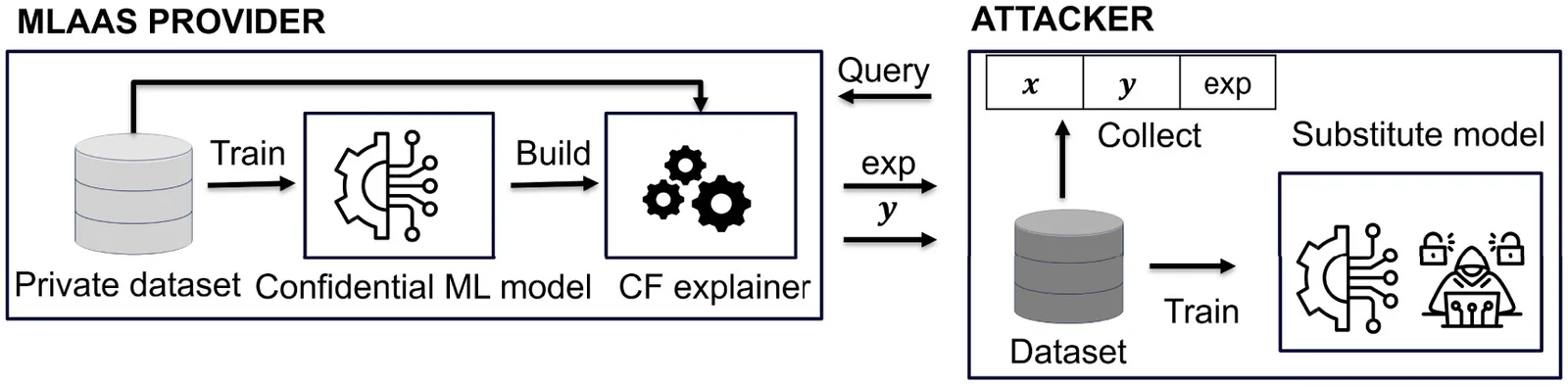
Scenario of MEA where MLaaS provides explanations alongside the prediction.

Figure 2 illustrates the steps of the proposed scenario. First, a user submits a query, containing input data representing a data record *x*, for which they seek a prediction. Upon receiving the query, the MLaaS API forwards it to the ML service, which computes the prediction *f*_θ_(*x*) and generates a corresponding CF explanation *c* = *E*(*x*), where *f*_θ_(*c*) yields a different prediction. Finally, the service returns both the prediction and the CF explanation to the user.

Within the scope of this work of this research, our primary emphasis is placed on a specific type of attack known as the high-fidelity MEA, specifically directed toward CFs. The objective of this attack is to derive a model threat_model *t*_Υ_ that accurately replicates the functionality of the target model.

The MEA is formulated as follows: for a set of queries Q and a set of corresponding CF explanations C, an attacker trains a threat model *t*_Υ_ that performs equivalently on an evaluation set T. The goal is to maximize an agreement function (the agreement metric will be detailed later in this paper in Section 5), between *f*_θ_ and *t*_Υ_ as shown in Equation 5, with a minimal number of queries sent to the API.

We now describe our proposed KD-based MEA and train a threat model *t*_Υ_. Then, we describe our proposed methods for integrating DP within the CF generation process.

**Figure 3:**
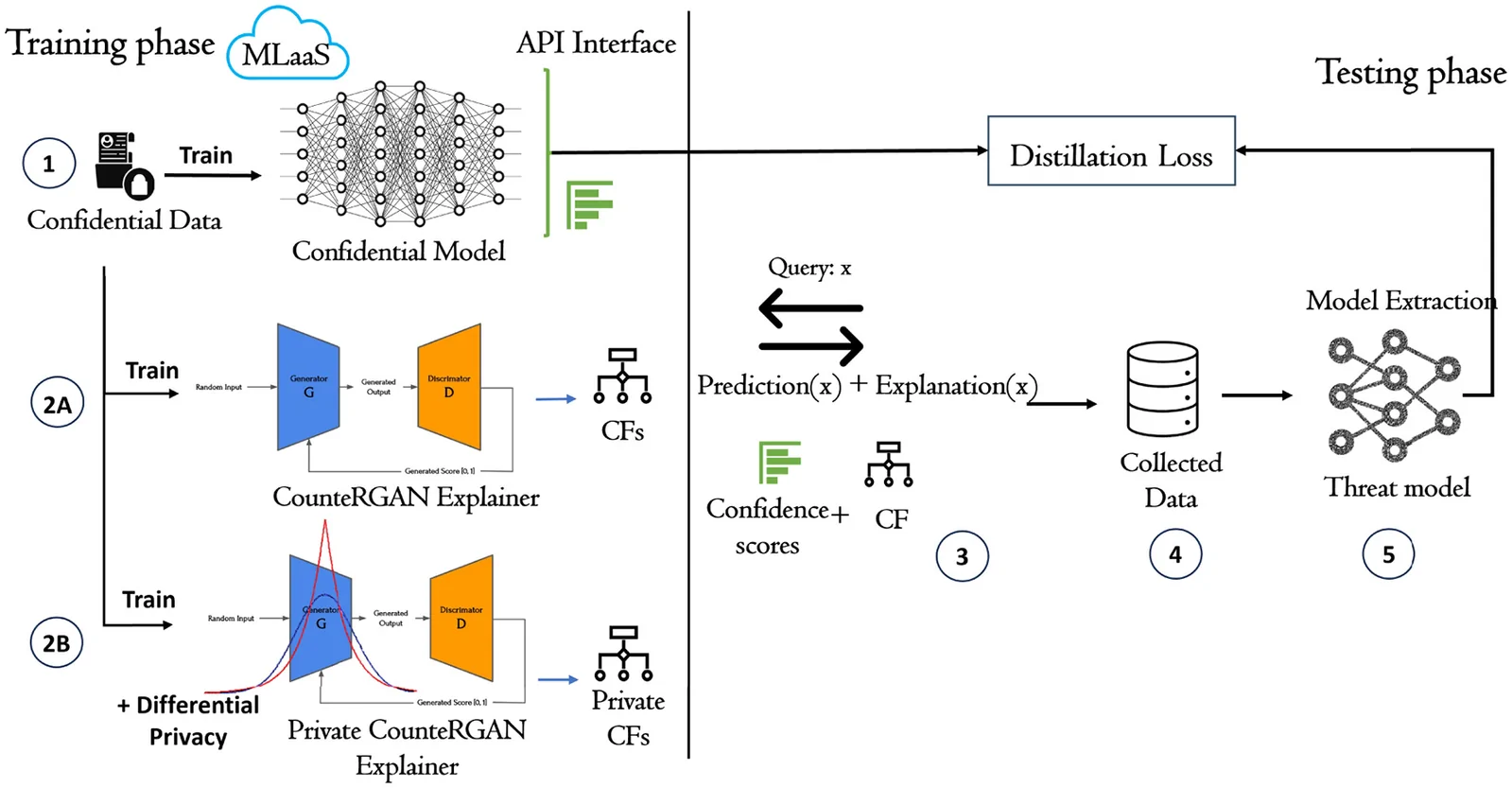
Methodology Framework, describing the KD-based MEA, in the deployment phase, in addition to the CF generation phase.

Figure 3 illustrates our proposed methodology of the KD method along with the CF generation process. An owner of a private dataset trains a DNN classifier (Step 1 in Figure 3), and a CounteRGAN CF generator (Steps 2A, 2B in Figure 3) and deploys it on an MLaaS platform.

To make the MEA more challenging for the attacker, we assume that the attacker does not have previous knowledge of the training set. The attacker generates random samples and collects both the query output of the target model and the corresponding CFs provided for those outputs (Step 3 in Figure 3). After successfully collecting a set of CFs (Step 4 in Figure 3), the attacker employs our KD-based MEA approach on the collected dataset as input data to train *t*_Υ_. The rationale for selecting KD as the extraction method lies in its unique capability to address the problem not only as a standard supervised classification task but also in its proficiency in precisely imitating the probability distribution. Specifically, we propose to train *t*_Υ_ by minimizing the loss constituted by the threat model classification loss in addition to the distillation loss. To emphasize the importance of mimicking the output probabilistic distribution from the target model to the threat model, we set the distillation loss to the Jensen-Shannon (JS) (Equation 4) metric. The choice of JS is to tackle issues related to divergence and ensure a symmetric output, so we suppose that the attacker replaces the KL divergence metric with JS. Afterward, the attacker trains the threat model by minimizing the loss of KD (Step 5 in Figure 3) until the convergence of the agreement on a separate validation set.


$$\begin{array}{l} \text{JS}\left(P\|Q\right)=\frac{1}{2}\text{KL}\left(P\|\frac{P+Q}{2}\right)+\frac{1}{2}\text{KL}\left(Q\|\frac{P+Q}{2}\right) \end{array}$$ (4)

### 4.1 Private CFs generation with DP

We are now aligned with the service provider and are presenting a strategy to mitigate MEA by providing private CFs through integrating DP into the CF generation process. Our methodology does not expose statistical information about the training set.

To this end, we integrate DP into the employed CF generator, CounteRGAN, during the optimization process as follows. First, we employ the Adam differential private optimizer (DP Adam). The process of DP Adam often involves multiple iterations of the training process to achieve privacy guarantees. We then add noise repeatedly over several rounds, such that the overall privacy guarantees are strengthened by injecting noise into the gradients of the generator in addition to a step of gradient clipping. This process involves clipping the gradient for a random subset of examples, clipping the norm of each gradient, computing the average, adding calibrated noise, and finally performing the traditional back-propagation step that updates the weights. Note that the gradient clipping step is essential as it controls the amount of noise added and prevents large updates that could reveal too much information about individual data points. The key assurance provided by this approach is that the generator ensures that it does not memorize or reveal details of the training data and therefore does not generate CFs that are very similar to the training points. While DP guarantees ensure the privacy of the CFs, the added noise can degrade their quality and affect the training process. We also aim to examine the effects of incorporating DP into the CF generation process on MEA performance and the quality of the provided CFs, considering metrics such as proximity, prediction gain, and plausibility (described later in Section 5.6), as DP has an impact on performance-utility trade-off and explainability-utility as well (Saifullah et al., 2024). Additionally, we aim to demonstrate how private CFs fit with the distribution of the training set.

## 5 Experimental settings

### 5.1 Datasets

We perform our experiments using three classification datasets, namely, *Give Me Some Credits* (Give Me Some Credit Dataset, 2024), *Credit Card Fraud* (Credit Card Fraud Dataset, 2024), and *Housing* (California Housing Dataset, 2024):

- Give Me Some Credits (GMSC) (Give Me Some Credit Dataset, 2024). The dataset is collected to forecast the likelihood that an individual will experience financial distress over the next 2 years, based on their financial and demographic information. The dataset comprises 150,000 applicants, of whom 139,974 are classified as good and 10,026 as bad. We follow the preprocessing described in [69]. Specifically, starting from the raw training data, we first build a balanced dataset by retaining all minority-class samples and downsampling the majority class to match their counts, resulting in 20,052 instances with 10,026 samples per class. We impute missing values in MonthlyIncome and NumberOfDependents, add quantile-binned versions of selected variables, and apply a preprocessing pipeline that includes median imputation with indicator variables for missing values, power transformation, feature expansion, and feature selection, resulting in 73 features as input.
- Credit Card Fraud (Credit) (Credit Card Fraud Dataset, 2024). The dataset contains transactions conducted by cardholders using credit cards in September 2013, represented by 30 features. In this dataset, transactions over two days reveal 492 fraudulent instances out of a total of 284,807. In the preprocessing pipeline, the Amount feature is scaled using RobustScaler, and the Time feature is normalized to the [0,1] range. We then construct a balanced dataset by retaining all fraud transactions and randomly sampling an equal number of non-fraud transactions, yielding approximately 1,000 instances with a balanced class distribution.
- Housing (California Housing Dataset, 2024). The dataset was created from the 1990 U.S. Census, have 20,640 samples consisting of eight features and a target variable representing the median house value for California districts, in dollars. The target variable is then split into two classes using a threshold set to the median. Specifically, we convert the original regression target into a binary classification task by thresholding at the median target value. This produces a balanced binary target. In the current implementation, the input features are standardized using the feature-wise mean and standard deviation.

### 5.2 Baseline scenarios

We compare our proposed approach for performing MEA, KD-based MEA, to 2 state of the art baseline approaches, presented in Aïvodji et al. (2020), referred to as *Direct Train* and Wang et al. (2022) referred to as *DualCF*. Direct Train trains a threat model as a standard supervised classification task using the raw training data, without incorporating any specific optimization techniques for MEA, and DualCF trains a threat model using the CF of the CF directly. We examine three scenarios pertaining to the MLaaS provider's inclusion or exclusion of CF explanations alongside the predictions of the ML model, 1) *No CF:* In this scenario, the MLaaS does not provide the user with any CFs. 2) *CF:* In this case, the MLaaS provides CFs using counteRGAN. *3) Private CF:* The MLaaS offers private CFs by implementing our proposed approach for generating private CFs.

We begin by conducting an analysis aimed at 1) demonstrating how CFs can be exploited for MEA and (2) assessing how our proposed KD-based approach can further strengthen MEA. We also 3) evaluate private CFs under both direct training and KD. To perform this analysis, we simulate the following six scenarios:

- *Direct No CF*. We train a model directly on the randomly generated data points, and the focus shifts to examining the potential of direct queries and analyzing the power of CFs and KD.
- *Direct CF*. The MEA is performed following the baseline (Aïvodji et al., 2020). We train a model directly on CFs, this dimension is dedicated to evaluating the standalone power of CFs without the incorporation of KD, providing insights into the capabilities of CFs separately.
- *KD-based No CF*. We employ our proposed KD-based approach on the randomly generated data points, and the focus shifts to examining the potential enhancement when KD is utilized independently of CFs.
- *KD-based CF*. We employ our proposed KD-based approach, trained with CFs, to analyze the influence of KD when applied alongside CF.
- *Direct Private CF*. We train a model directly on CFs extracted from the DP generator, the focus shifts to examining the potential of direct queries and to analyzing the power of KD and CFs alongside.
- *KD-based Private CF*. We employ our proposed KD-based approach on CFs extracted from the differentially private generator, and the focus shifts to examining the potential effect of integrating DP in the CF generation.

We then extend our analysis to the second baseline, *DualCF*. These scenarios are designed to (1) evaluate the standalone effectiveness of DualCFs as a baseline compared to our KD-based approach, and (2) investigate the impact of integrating DualCFs with KD, yielding four distinct scenarios:

- *DualCF*. We train a model directly on DualCF, the aim is to evaluate the standalone power of DualCFs compared to KD, providing insights into their capabilities separately.
- *Private DualCF*. We train a model directly on DualCFs extracted from the DP generator, the focus shifts to examining the effect of DP and to analyzing it with DualCFs alongside.
- *KD-based DualCF*. We employ our proposed KD-based approach, trained with DualCFs, to understand the influence of KD when applied alongside DualCF.
- *KD-based Private DualCF*. We employ our proposed KD-based approach on DualCFs extracted from the DP generator, the focus shifts to examining the potential effect of integrating DP on the MEA.

To better represent the experimental design and to compare scenarios before examining the numerical results, Table 1 explicitly lists, for each scenario, the queried input source, the data used to train the extracted model, whether CFs are used, whether the CFs are private, whether KD is used, and which extraction family the scenario belongs to.

**Table 1:** Summary of experimental scenarios used in the MEA evaluation.

Scenario	CF	Private CF	DualCF	KD	Direct
Direct No CF	×	×	×	×	✓
Direct CF	✓	×	×	×	✓
KD-based No CF	×	×	×	✓	×
KD-based CF	✓	×	×	✓	×
Direct Private CF	×	✓	×	×	✓
KD-based Private CF	×	✓	×	✓	×
DualCF	✓	×	✓	×	✓
Private DualCF	×	✓	✓	×	×
KD-based DualCF	✓	×	✓	✓	×
KD-based Private DualCF	×	✓	✓	✓	×

### 5.3 Target model

We adhere to consistent training procedures for the target model (to be deployed and queried by the API). We train a DNN comprising of 16 hidden layers to create a complex model. The layer configurations consist of 64, 32, 16, 32, 64, 128, 64, 32, 128, 64, 128, 64, 128, 64, 32, and 16 neurons in each layer, respectively. The activation function is set to Gelu for all layers, while the output layer employs softmax to produce model outputs as confidence score probabilities. We specify the Adam optimizer to minimize the cross-entropy loss function, with default parameter initialization. Each target model is trained using 80% of the training dataset. For all datasets, the model weights were initialized using LeCun uniform initialization with a random seed, a dropout rate of 0.2, and the Adam optimizer with a learning rate of 0.05, a batch size of 32, and 1000 training epochs.^2^

### 5.4 Threat model

We specify consistent training procedures for the threat model, assuming that attackers have no knowledge of the target model architecture but can build a DNN with a standard architecture. The attacker aims to train the threat model either using our proposed KD-based MEA approach or directly on the data points, using a DNN with three hidden layers of 16, 32, and 64 neurons consecutively, with ReLU activations and a softmax output layer. In the scenario we are considering, we assume that the attacker does not possess any knowledge about the distribution of the training data used for training the target model. Consequently, the attacker generates 1000 random data points for each dataset. The data point values are randomly generated, with values drawn from a range of -3 to 3 for each feature. We specify -3 and 3 as the ranges for random values, aiming to obtain randomized data points that are as dissimilar as possible to the training datasets. The threat model is trained for 1000 epochs, Adamax as optimizer with a learning rate of 0.01. For the KD-based MEA training, we vary α from 0 to 0.5 and report the highest agreement across 1000 epochs. We report the average of 10 runs with randomly selected subsets for each experiment.

### 5.5 Counterfactual generator

To generate CFs using CountRGAN, we train the discriminator with 3 layers comprising 32, 16, and 1 neurons, respectively. Each hidden layer is followed by a dropout layer with a factor of 0.2. We use a ReLU activation function for the hidden layers and a sigmoid activation function for the output layer. The CountRGAN schedule consists of two discriminator steps and four generator steps, with a total of 2000 training iterations and a minibatch size of 64. The generator, which takes a datapoint as input and produces the corresponding CF, is trained with three hidden layers comprising 64, 48, and 32 neurons, using ReLU activation for the hidden layers and a linear activation function for the output layer. We specify the optimizer as Adam with a learning rate of 0.005. For the DP generator, we change the optimizer to one that supports DP. We integrate the DP Adam optimizer from the TensorFlow privacy library. To ensure a good privacy budget, we set the DP Adam optimizer's l2_norm_clip to 1 and the noise_multiplier to 3.

### 5.6 Evaluation metrics

The evaluation metrics comprise two sets. The first set measures the effectiveness of MEA while the second set of metrics asses the quality of the CFs.

**Effectiveness of MEA**: A commonly employed metric for assessing the effectiveness of MEA is the *Agreement* measure. *Agreement* measures the degree of alignment between two models, i.e., the similarity in the predictions between the two different ML models. Agreements assess the output similarity between the predictions of an extracted model *t*_Υ_ and those of the target model *f*_θ_ for a given set of data records (see Equation 5). Since our goal is to replicate the behavior of a target model with an extracted model, a higher agreement means a more successful MEA, and hence, a more effective strategy for MEAs.


$$\begin{array}{c} agreement\left(f_{\theta },t_{\Upsilon }\right)=\underset{x_{i}\in T}{\sum }1_{f_{\theta }=t_{\Upsilon }} \end{array}$$ (5)

**Success of explainer and quality of CFs**: To measure the quality of extracted CFs, and to analyze the CF generator prediction classes shift we employ three commonly used metrics, namely, *Prediction Gain, Plausibility*, and *Proximity*.

The *Prediction Gain* (Equation 6) measures the change in the classifier's predicted probability for a specific target class *t* when comparing the CF explanation to the original data point. In other words, it quantifies the extent to which the generator succeeds in shifting the classifier's prediction toward the target class using the generated CFs. In our case, as the classifier outputs probabilities, the Prediction Gain ranges from 0 to 1, with higher values indicating a stronger shift toward the target class *t*.


$$\begin{array}{c} PredictionGain=f_{\theta }\left(t|CF\right)-f_{\theta }\left(t|input\right) \end{array}$$ (6)

*Plausibility* quantifies how closely a data instance fits a known data distribution. In this work, we leverage DP to prevent CFs from revealing statistical information about the training set. Accordingly, realism as a plausibility measure assesses how well both standard and private CFs conform to the underlying data distribution. Realism also enables comparison between a data point and its corresponding CF to evaluate the authenticity of CFs and the effect of DP-induced perturbations. Following Nemirovsky et al. (2022); Joshi et al. (2019), we train a denoising autoencoder on the noised training set and compute realism as the reconstruction error, measured by the mean squared error of the autoencoder (Equation 7). Lower realism values indicate a closer fit to the data distribution and better plausibility.


$$\begin{array}{c} Realism=\frac{1}{N}\overset{N}{\underset{i=1}{\sum }}\|input_{i}-reconstruction_{i}{\|}^{2} \end{array}$$ (7)

*Proximity* is a commonly used metric that allows for measuring the quality of a CF. It considers both the number of altered features and the magnitude of those changes (amount of modification in a feature) relative to the original data instance. We compute *Proximity* by taking the L1 norm of the absolute difference between the data point and its corresponding CF (Equation 8). Lower *Proximity* values (desired) indicate that only a small subset of input features has been perturbed to achieve a different outcome, suggesting higher-quality CFs.


$$\begin{array}{c} \text{Proximity}=\frac{1}{N}\overset{N}{\underset{i=1}{\sum }}\|{\text{input}}_{i}-{\text{CF}}_{i}{\|}^{1} \end{array}$$ (8)

By computing this comprehensive set of metrics, we aim to explore how DP impacts the feasibility of taking actionable steps and how this influence contributes to the prediction gain outcomes.

## 6 Numerical results

### 6.1 Agreement for model extraction attack

We start our discussion by analyzing the *agreement* obtained by the employed approaches for MEA. During the training phase of the threat model, the attacker interacts with the target model by querying it with randomly generated data points. In our experimentation, we systematically increase the number of queries between 50 and 1000 to conduct a comprehensive analysis of their impact on MEA.

**Figure 4:**
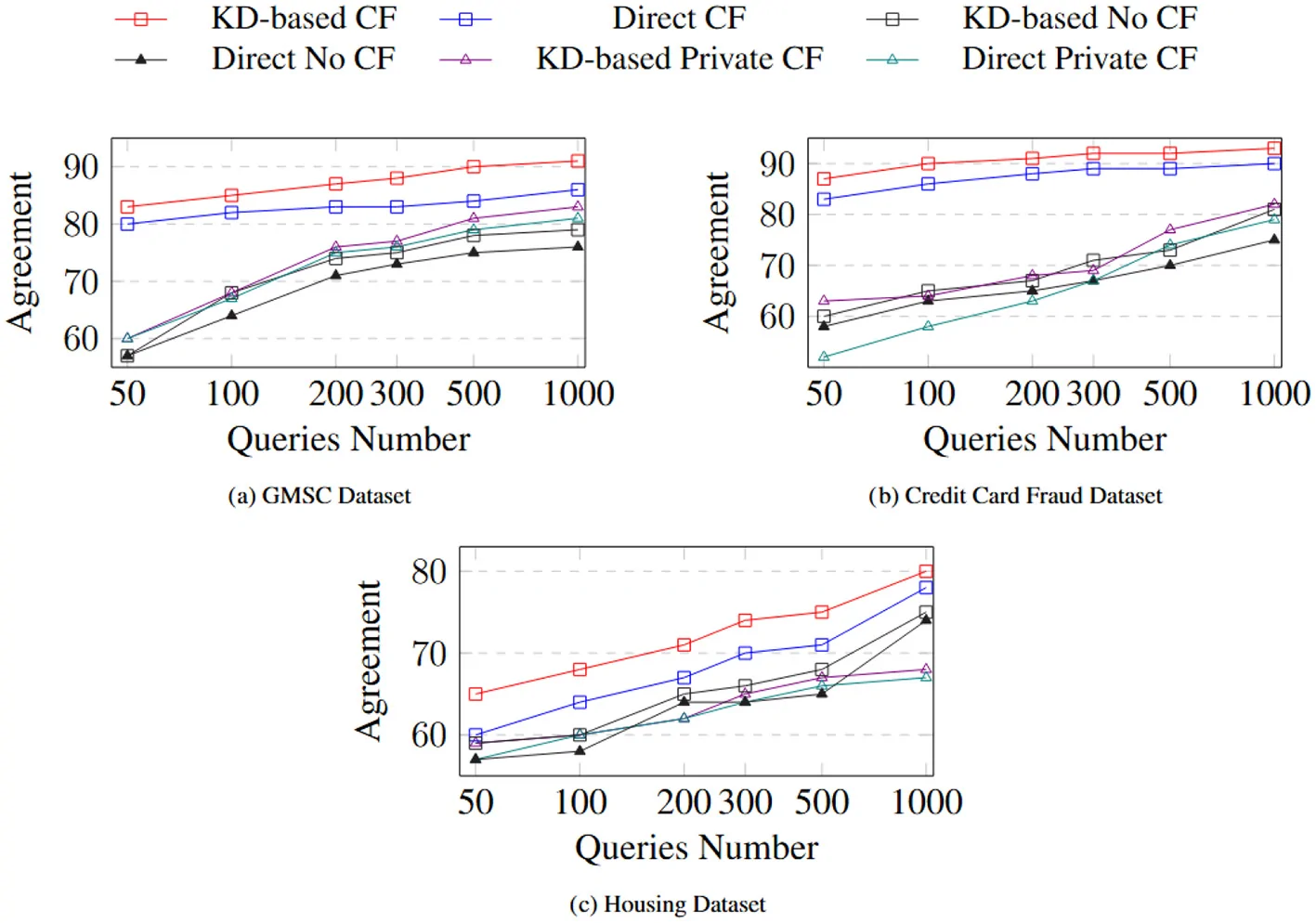
The agreement values achieved by the MEA approach in the various scenarios across the **(a)** GMSC dataset, **(b)** Credit Fraud Dataset, **(c)** Housing Dataset with respect to number of queries made.

Figures 4a–c show the *agreement* achieved by the six scenarios (i.e., with *KD-based MEA* and *Direct* as MEA methodology, and while leveraging CFs, private CFs or without CFs), with respect to the number of queries made to the API across the GMSC, Credit Card Fraud, and Housing datasets, respectively. First, we notice that results show a consistent pattern across all scenarios, as *agreement* initially increases significantly with the number of queries until a point where additional queries cease to yield a notable increase. Note that this pattern is less noticeable for *KD-based CF* and *Direct CF*, which already exhibit relatively high *agreement* even with a low number of queries. As a result, these methods show a less substantial improvement in *agreement* compared to the others. We analyze deeper this observation in the following discussion. Nevertheless, *Takeaway 1: Additional Queries lead to a saturation point in the MEA, up until further queries may not necessarily contribute to conducting a more effective MEA, which is in line with the findings reported in the literature*.

**We now focus on analyzing the extent to which CFs can strengthen the adversary's ability to perform MEAs**, comparing the *agreement* achieved by each of *KD-based MEA* and *Direct*, when CFs are and are not utilized (i.e., comparing *Direct No CF* to *Direct-CF*, and *KD-Based No CF* to *KD-based CF*). This comparison focuses on the use of CFs for MEA, independent of the methodology applied. In both cases, when CFs are used, and across all three datasets, the MEA is significantly more effective, achieving an *agreement* higher than its counterpart. For instance, in GMSC, *Direct-CF* achieves an *agreement* ranging between 80 and 85, up to 25% in some cases than *Direct No CF*, which achieves an *agreement* ranging between 55 and 75 (never higher than that of *Direct-CF*). For Credit Card Fraud, this difference is further amplified when CFs are used, as *Direct-CF* shows an *agreement* ranging between 83 and 90 while *Direct-no-CF* achieves an *agreement* ranging between 58 and 75. Similar results are observed across the Housing, where exploiting CFs allows for an additional agreement of around 10%. Moving to *KD-based MEA*, i.e., comparing *KD-based CF* to *KD-Based No CF*) across the three datasets, we notice that exploiting CFs permits achieving an agreement of up to 27% more (for GMSC at 50 queries) and, in the worst case, an additional 4% on *agreement* (for Housing at 1000 queries), than when CFs are not used. *Takeaway 2: Utilizing CF has a significant impact on leveraging model's CF for performing MEA regardless of the methodology used (KD-based MEA or Direct)*.

**We now switch to comparing the effectiveness of our proposed approach (*KD-based MEA*) to that of** ***Direct*** across the three cases of leveraging CFs or not (i.e., no CF, CF, and Private CF), across the three datasets. We start with the case where no CFs are used. Results show that across all datasets, *KD-based MEA* outperforms its *Direct* counterpart. More specifically, *KD-based No CF* maintains a 3% to 6% higher *agreement*, across all datasets, than *Direct no CF*. Another way of looking at the performance is in terms of number of queries required by each approach to achieve a particular performance, which allows to understand better the effectiveness of a given approach. In this consideration, using CFs significantly reduces the number of instances required for MEA to reach a specific high agreement level. For example, across GMSC, achieving 80% agreement with CFs took just 50 queries, while accomplishing the same level without CFs required over 1,000 queries. This pattern holds across datasets, such as the Credit Card Fraud Dataset, where 83% agreement needed only 50 CF queries compared to over 1,000 queries without CFs. *Takeaway 3: The use of CFs consistently leads to a substantial reduction in the number of queries needed to achieve high agreement levels across diverse datasets*.

We now consider the case where CFs are used (i.e., *KD-based CF* vs. *Direct CF*) with the aim of analyzing the extent to which KD-based approach strenghten the MEA Results show that across all datasets, and for any number of queries, *KD-based CF* is more effective than *Direct CF*, maintaining an *agreement* of 2% to 6% higher than that of *Direct CF*. Investigating these results in terms of number of queries, we notice that *KD-based CF* can be significantly more effective than *Direct CF*. Specifically, *KD-based CF* achieves an *agreement* of 82% and 85% with only 50 and 100 queries, a performance that is achievable by *Direct CF* with x6 number of queries (300) and x10 number of queries (500), respectively. A similar finding can be extracted across the Credit Card Fraud, as KD-based CF achieves an *agreement* of 90% with only 100 queries while *Direct-CF* uses 1,000 queries to attain such an effective MEA. Similar results can also be observed analyzing the performance achieved by these two scenarios with the Housing. *Takeaway 4: The KD-based MEA consistently results in higher agreement levels of MEA compared to the baseline approach. This holds in various scenarios and is independent of whether the MLaaS provides users with CF explanations, and across diverse datasets and query numbers. Importantly, when CFs are employed, MEA requires significantly fewer instances to reach a specific high agreement level. This highlights the efficiency of incorporating CFs in conjunction with KD to enhance the performance of the MEA*.

The experimental results and takeaways collectively highlight the interplay between CFs and KD within the context of MEA. A central observation is that the KD-based MEA consistently outperforms the baselines when CFs are exploited. This behavior aligns with the theoretical strengths of KD, where soft probability distributions provide a rich representation of training datapoints than hard labels, and CFs, by construction, lie close to the decision boundary, where these soft representations are most informative. As a result, the extracted model accurately reconstructs the target model's decision-making, leading to high agreement and improvements across datasets and query budgets.

We now turn our attention to the scenario in which the MLaaS provider offers users private CFs, i.e., comparing *KD-based Private CF* to *Direct Private CF*.

**We analyze the impact of generating private CFs through integrating DP in the CF generator**, i.e., by incorporating DP during the generation process of explanations, on mitigating MEA. This analysis aims to quantify the degree of protection offered by providing users with private CFs (as opposed to CFs that are not generated with DP) such as to prevent attackers from leveraging vulnerabilities associated with non-private CFs. To this end, we compare the performance of each of *KD-based CF* and *Direct CF* to its private CF counterparts (*KD-based Private CF* and *Direct Private CF*, respectively). In GMSC, results show that for the relatively low number of queries (50 to 200), the difference is vast between each scenario with private CF and its counterpart. For instance, *KD-based Private CF* achieves an agreement ranging between 60% and 75% while that *KD-based CF* obtains an agreement between 83% and 87%. We observe that the incorporation of DP shows that our proposed strategy can maintain agreement levels comparable to scenarios without CFs and less than when incorporating traditional CFs, with an agreement range of 60% to 83% with *KD-based CF* and 60% to 81% with direct training, compared to 57% to 79% with *KD-based MEA no CF* and a range of 57% to 76% with *Direct No CF*. For the credit card fraud dataset, a similar trend is observed. With *KD-based Private CF*, the agreement range is between 63% and 82% and 52% and 79% for a direct-DP train, compared to *KD-based no CF* of 60% to 81% and a range of 58% to 75% with *Direct No CF*. For the housing dataset, the agreement of *KD-based CF* ranges from 59% to 68% with KD and 57% to 67% with *Direct Private CF*, compared to 59% to 75% with *KD-based no CF*, and direct no CF ranges from 57% to 74%. It is also worth noting that *KD-based Private CF*is slightly better than direct training over all datasets. For *KD-based MEA*, when DP is integrated, the noise introduced during the training process to achieve privacy guarantees could interfere with the teacher-student model's ability to transfer knowledge much more effectively. The observed lower in agreement of MEA with private CFs may be attributed to the privacy-preserving nature of DP. DP introduces intentional noise into the data to protect individual privacy, making it more challenging for attackers to accurately extract sensitive information, since CFs may no longer be very close to the decision boundary. *Takeaway 5: The incorporation of DP can play a crucial role in maintaining agreement levels comparable to scenarios without CFs, as they show that across the three datasets, a mitigation can be achieved*.

Takeaways show that when DP is introduced into the CF generation process, the behavior changes substantially. DP injects calibrated noise into the gradients of the CF generator, which impacts the generated CFs. This degradation manifests in lower plausibility with respect to the training distribution and increased perturbation variance (discussed later in details). These distortions weaken the alignment between CFs and the true decision boundary, thereby impacting the effectiveness of KD. More specifically, because DP enforces privacy by bounding the sensitivity of each gradient update, it systematically suppresses fine-grained, instance-level information, precisely the information CF generators rely on to produce minimal, directionally accurate perturbations.

We now turn to the results of the second MEA baseline, *DualCF*, comparing it with our proposed *KD-based* approach in terms of agreement, including evaluations involving Private CFs. To reduce redundancy and highlight the most relevant insights, we limit our presentation to query sizes of 200 and 1,000. Table 2 presents the agreement of the *Direct CF, DualCF, KD-based CF, KD-based DualCF*, along with their respective scenarios involving Private CFs.

**Table 2:** Agreement values achieved by the MEA approach in comparison with DualCF baseline scenarios across the **(a)** GMSC, **(b)** Credit Fraud, and **(c)** Housing datasets, with respect to the number of queries made.

Dataset	Query	Direct CF	Dual CF	KD-based CF	KD-based DualCF	Direct private CF	DualCF private	KD-based private CF	KD-based private DualCF
Housing	200	67	70	71	79	62	62	62	71
	1000	78	79	80	82	67	68	68	76
Credit fraud	200	88	89	91	92	63	64	68	69
	1000	90	90	93	93	79	80	82	85
GMSC	200	83	83	89	89	75	77	76	82
	1000	86	87	91	92	81	82	83	84

We first compare the MEA agreement of *KD-based CF* to *DualCF*, across all datasets and query sizes. *KD-based CF* demonstrates clear improvements over *DualCF*: on Housing, agreement reaches 71% vs. 70% with 200 queries and 80% vs. 79% with 1,000 queries. On Credit Card Fraud, 91% vs. 89% with 200 queries and 93% vs. 90% with 1,000 queries. Similar trend on GMSC, 89% vs. 83% with 200 queries and 91% vs. 87% with 1,000 queries. With regard to *KD-based Private CF*, it maintains equivalence or superiority in nearly all cases. On Housing, both methods yield identical agreement at 62% with 200 queries and 68% with 1,000 queries. On Credit Card Fraud, *KD-based Private CF* leads with 68% vs. 64% for *DualCF Private* at 200 queries and 82% vs. 80% at 1,000 queries, respectively. On GMSC, *KD-based Private CF* slightly outperforms *DualCF Private* at 1,000 queries with 83% vs. 82%, with only one exception at 200 queries where *DualCF Private* attains 77% and *KD-based Private CF* reaches 76%. *Takeaway 6: KD produces more accurate and generalizable MEA by effectively capturing underlying fidelity and structural patterns in the data compared to DualCF, as shown across multiple datasets, query sizes, and privacy conditions, and evidenced by higher agreement scores*.

We now analyze the *agreement* of *KD-based CF* compared to *KD-based DualCF*, and *KD-based Private CF* compared to *KD-based Private DualCF*. A consistent trend is evident across all datasets and query sizes: integrating *DualCF* with KD further increases *agreement* relative to *KD-based CF*. In the absence of DP, *KD-based DualCF* achieves the highest overall performance. For the Housing dataset, agreement improves from 71% with *KD-based CF* to 79% with *KD-based DualCF* at 200 queries, and from 80% to 82% at 1,000 queries. On the Credit Card Fraud dataset, *agreement* increases from 91% to 92% at 200 queries and remains at 93% at 1,000 queries. For GMSC, *agreement* remains at 89% at 200 queries and increases from 91% to 92% at 1,000 queries. The same trend is observed for *KD-based Private DualCF* compared to *KD-based Private CF*. On Housing, *agreement* improves from 62% with *KD-based Private CF* to 71% with *KD-based Private DualCF* at 200 queries, and from 68% to 76% at 1,000 queries. On Credit Card Fraud, *agreement* increases from 68% to 69% at 200 queries and from 82% to 85% at 1,000 queries. Similarly, on GMSC, *agreement* improves from 76% to 82% at 200 queries and from 83% to 84% at 1,000 queries. These results demonstrate that integrating *DualCF* with KD consistently enhances *agreement* across all datasets and query sizes, with particularly notable improvements under DP. *Takeaway 7: Combining KD with DualCF yields consistently higher agreement over KD-based CF alone, across all datasets, query sizes, and privacy settings. This combination leverages the representational strength of KD while benefiting from the structural alignment of DualCF, resulting in enhanced agreement and MEA*.

We move to analyzing the effect of query size on the *agreement* of *DualCF, KD-based DualCF* across the three datasets. A consistent trend emerges: agreement tends to be stronger with larger query counts across all datasets and privacy scenarios. For example, with *DualCF, agreement* reached 62% with 200 queries and 68% with 1,000 queries on the Housing dataset, from 64% to 80% on credit card fraud, and from 77% to 82% on GMSC. A similar trend is evident in the Private Scenarios: *agreement* of MEA on Housing improves from 70% to 79%, Credit Card Fraud from 89% to 90%, and GMSC from 83% to 87%. These findings suggest that larger query sizes enhance agreement for *DualCF*.

We proceed to compare the *agreement* of *DualCF* to *DirectCF*, and *DualCF Private* to *Direct Private CF*, across the two query sizes on all datasets. As expected, the results consistently indicate that *DualCF* outperforms *DirectCF* across all evaluated scenarios. Similarly, *DualCF Private* demonstrates superior performance relative to *Direct Private CF*, under privacy constraints. For instance, on the Housing dataset, *DualCF* achieves 70% *agreement* compared to 67% for *DirectCF* at 200 queries, and 79% vs. 78% at 1,000 queries. On the Credit Card Fraud and GMSC datasets, *DualCF agreement* either matches or exceeds *DirectCF*: at 1,000 queries, both methods yield 90% *agreement* on Credit Card Fraud, while GMSC shows a marginal advantage for *DualCF* (87% vs. 86%). These findings are consistent with prior results reported in the original research in Wang et al. (2022), reinforcing the conclusion that *DualCF* is a more effective MEA approach than *DirectCF* across varying query sizes and privacy conditions.

From a security and privacy perspective, these findings reveal that DP alone does not eliminate the risk of MEA facilitated by explanations. Instead, DP reduces the attack surface but remains vulnerable when attackers can issue many queries. This suggests that MLaaS providers should consider DP as one component of a broader defense strategy, complemented by rate limiting or query auditing.

### 6.2 Impact of incorporating DP in CF generator on quality of explanations

**Figure 5:**
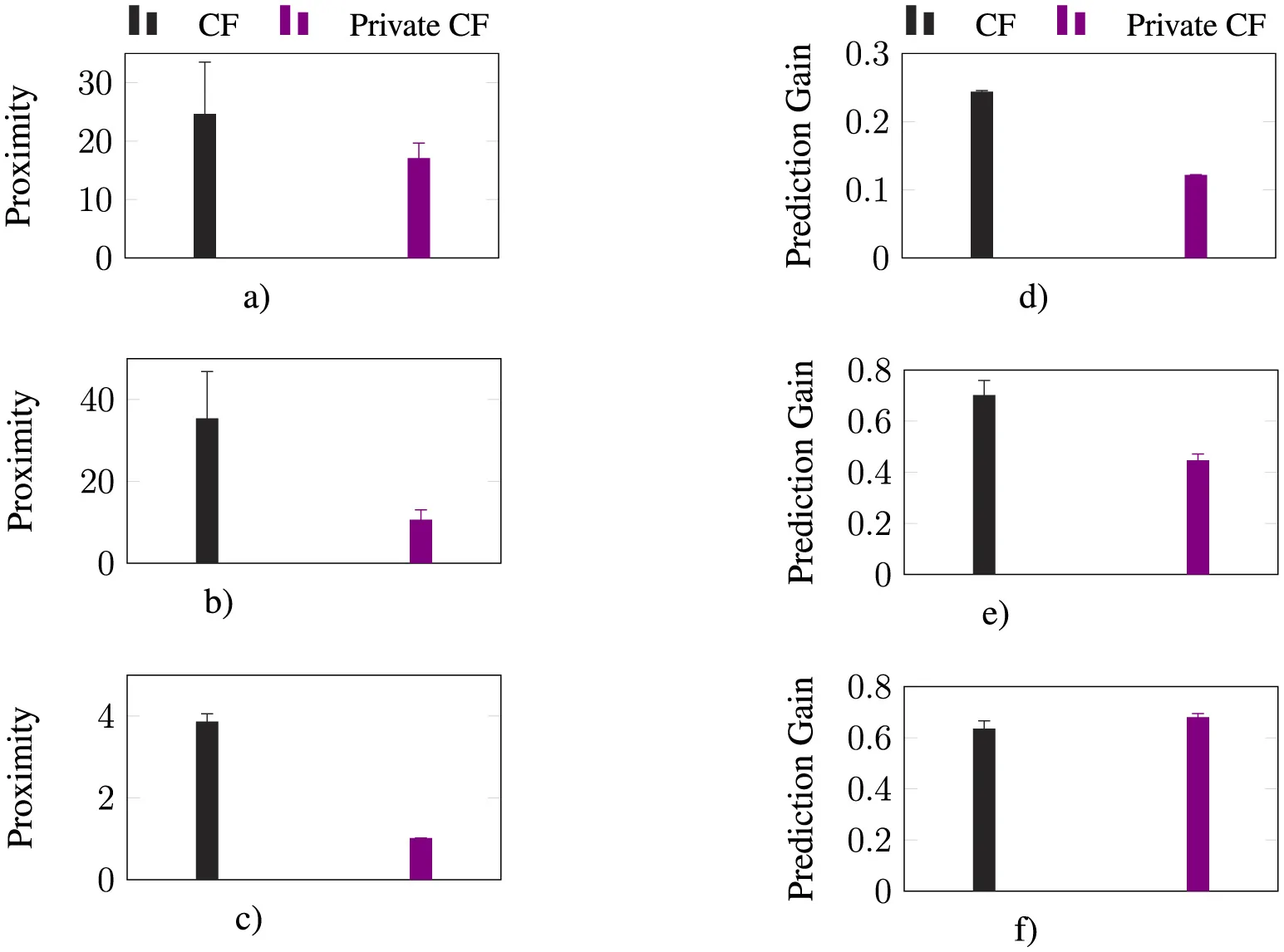
Proximity and prediction gain (± std) for CFs and Private CFs across datasets Actionability is dataset dependant since the ranges are based on the feature values. **(a, d)** GMSC. **(b, e)** Credit fraud. **(c, f)** Housing.

We now switch to analyzing the impact of integrating DP into CF generation process, i.e., into the CounteRGAN CF generator, on the quality of CFs in terms of the metrics introduced in Section 5.6.^3^ Figure 5 shows CounteRGAN's *proximity* and *prediction gain* of the generated CFs with and without incorporating DP, across the three datasets.^4^

In terms of *proximity*, which measures the degree of perturbation (or modification) of the CF compared to the initial point, results show that the generator generates CFs with lower values of *proximity* when DP is integrated than when not, across all datasets. For instance, for GMSC dataset, the *Proximity* of CFs with DP is 16.981 (± 0.158) while that without DP is 24.5 (± 0.364). Similarly, for Credit Fraud and Housing, *proximity* is lower when integrating DP from 35.269 ± 0.328 to 10.507 ± 0.238, and from 3.852 ± 0.053 to 1.004 ± 0.016, respectively. This means that that the practical usefulness of the CFs or perturbations in guiding actionable decisions is reduced, suggesting that the privacy constraints imposed might compromise the effectiveness of CF analyses in providing actionable guidance for influencing desired outcomes. *Takeaway 8: CFs produced with DP integration consistently exhibit lower perturbation (proximity) values compared to those without DP. This suggests that the integration of DP leads to CFs requiring fewer modifications to preserve privacy. We did not optimize or fine-tune the level of privacy, these results show/quantify the existing tradeoff between the extent we can protect privacy and impact the quality in terms of proximity*.

Results show that CounteRGAN achieves a higher *prediction gain* (a higher probability shift, desired) when DP is not integrated within the CF generator than when not for GMSC (0.243 ± 0.011 vs. 0.121 ± 0.01) and Credit Fraud datasets (0.700 ± 0.084 vs. 0.445 ± 0.06) while for Housing dataset, CounteRGAN shows a prediction gain slightly higher in the case where DP is integrated rather than when not (0.678 vs. 0.633). Unlike previous observations, the prediction gain of CFs and private CFs is comparable in this case. *Takeaway 9: The impact of DP integration on the prediction gain is dataset-specific, either maintaining it or, in some cases, restricting the explainer's progress toward the target class. This can be monitored and evaluated based on the use case and dataset*.

Although a higher prediction gain and lower proximity are preferable when generating CFs, the results suggest that the CF generation with DP has taken a different trajectory than it has taken without DP, leading to a reduction in prediction gain. This shift in the CF generation approach is reflected in the probability, ultimately contributing to the observed decrease in agreement, thereby motivating the smaller agreement for MEA.

**Figure 6:**
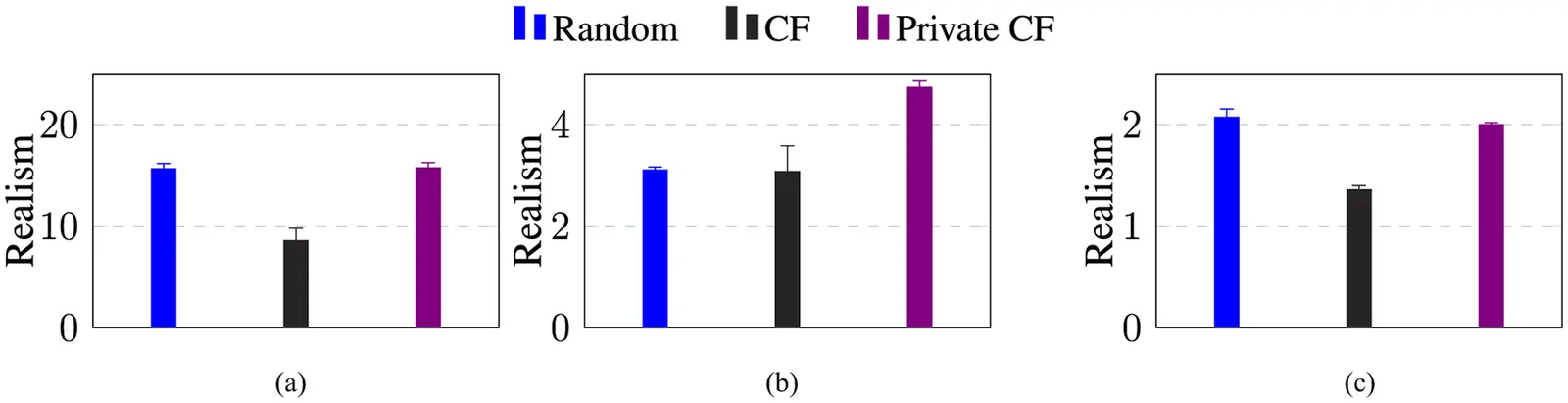
Realism scores (± standard deviation) for Random points, CFs, and Private CFs across the **(a)** GMSC, **(b)** Credit Fraud, and **(c)** Housing datasets.

Finally, we analyze the plausibility of the CFs in terms of *realism* of the data points, which enables us to quantify how well a data instance fits a data distribution of a dataset, including CFs in the scenarios with CF and with Private CF, and therefore to investigate the impact of incorporating DP in the generator. Combining this analysis with the previous ones, we can gain a deeper understanding of the impact of DP on MEA and the CFs. Figure 6 shows the *realism* of the data points used as initial queries and the CFs generated by the generator across the three datasets. We purposely present the initial queries *realism* to show the plausibility of the initial points compared to their corresponding CFs. Results show that *realism* of CFs when DP is not integrated in the generator is the lowest in comparison to that of initial queries and that of CFs when DP is employed. Specifically, in GMSC, the initially generated points have an average realism of 15.6 (± 0.03), and the CF generator has produced corresponding CFs with a realism of 8.5 (± 0.14). Similarly, in Credit Fraud, the Realism of initial data points is 3.104 (± 0.019) while that of the corresponding CFs is slightly lower at 3.07 (± 0.16), respectively. In Housing, the realism of initial data points is 2.07 (± 0.04) while their corresponding CFs had a realism of 1.35 (± 0.03). This indicates that the CF generator, when DP is not integrated, generates CFs with lower realism from the original queried random points and aligns them more closely with the distribution of the training data. *Takeaway 10: The CF generator without DP integration has produced more plausible data points (lower realism) which means that the CFs fit better with the distribution of the training data*.

As shown in Figure 6, initial random points are inherently unrealistic (do not exhibit high realism). Our private CF generation approach ensures this unrealistic nature is preserved when queried with a random data point. This is crucial because our experiments showed that traditional CFs, without privacy protections, generate more realistic outputs from random data, potentially revealing private information. Moreover, results also show that the private CFs preserve a realism very similar to that of random points. For instance, in GMSC, the private CFs have preserved their initial average level of realism of 15.723 (± 0.033). Similarly, in Housing, they preserve a realism of 2.0 (± 0.01). In Credit Fraud, the realism of the CFs deviates by one degree from the distribution, resulting in a realism of 4.73 (± 4.73). This means that incorporating DP, realism can be preserved indicating the effectiveness of the Private CFs in maintaining the quality and distribution of the original data. *Takeaway 11: The CF generator, with DP integration, guarantees that the generated CFs maintain a similar level of plausibility to the original query data points*.

### 6.3 Classical performance metrics

Table 3 reports the classification performance of the extracted models using our proposed KD-based MEA and the state-of-the-art approaches, Direct and DualCF, compared to the performance of the original models, across all datasets, query budgets, and DP privacy variants (evaluation performed on a separate test set).

**Table 3:** Accuracy, precision, recall, and F1-score for all scenarios across all datasets.

Dataset	Query	Metric	Original model	Direct CF	Dual CF	KD-based CF	KD-based DualCF	Direct private CF	DualCF private	KD-based private CF	KD-based private DualCF
Housing	200	Accuracy	88	66	70	70	79	61	62	62	72
		Precision	88	65	70	71	79	61	61	63	73
		Recall	88	64	70	70	78	60	61	62	72
		F1-score	88	64	70	71	78	61	61	62	72
	1000	Accuracy	88	77	79	80	82	67	68	68	76
		Precision	88	77	77	80	81	68	65	69	77
		Recall	88	77	78	80	80	66	66	68	76
		F1-score	88	77	77	80	81	67	65	68	76
Credit Fraud	200	Accuracy	91	88	89	91	92	63	64	68	69
		Precision	91	87	88	90	91	62	65	67	70
		Recall	91	89	90	92	91	64	63	69	68
		F1-score	91	88	89	91	91	63	64	68	69
	1000	Accuracy	91	90	90	93	93	79	80	82	85
		Precision	91	89	91	92	94	78	81	83	84
		Recall	91	91	89	94	92	80	79	81	86
		F1-score	91	90	90	93	93	79	80	82	85
GMSC	200	Accuracy	87	82	83	89	89	75	77	76	82
		Precision	88	81	84	88	90	74	78	77	81
		Recall	87	83	82	90	88	76	76	75	83
		F1-score	87	82	83	89	89	75	77	76	82
	1,000	Accuracy	87	85	87	91	92	81	82	83	84
		Precision	88	84	88	90	93	80	83	82	85
		Recall	87	86	86	92	91	82	81	84	83
		F1-score	87	85	87	91	92	81	82	83	84

The original model is trained on the original dataset and is reported as a separate baseline.

First, as expected, increasing the query budget from 200 to 1,000 improves the performance of all extraction approaches. This trend holds across accuracy, precision, recall, and F1-score, confirming that additional queries provide higher performance and better extraction for the deployed model. In other words, increasing the number of queries from 200 to 1,000 generally improves performance across the various MEA approaches, and the extracted models have comparable performance to the original model. Second, the KD-based approaches consistently achieve the highest performance across all metrics and datasets compared to Direct and DualCF. In particular, KD-based CF and KD-based DualCF, including its private variant, stand out as the most robust methods, maintaining superior predictive quality even under limited query budgets. This demonstrates that incorporating KD into MEA not only improves fidelity but also enhances the generalization ability of the extracted model. More importantly, the relative ranking of the approaches remains stable across scenarios. The KD-based approach, especially KD-based DualCF and KD-based Private DualCF, consistently achieves the highest performance, demonstrating that it is not only effective but also robust across datasets and query counts. Third, precision, recall, and F1-score also remain closely aligned with accuracy and reflect a consistent overall improvement in predictive quality. Interestingly, in some cases, the extracted model slightly outperforms the original model on ground-truth test labels. This is plausible because the regularization effect of distillation has been observed in prior KD teacher-student settings. However, this does not imply higher agreement (fidelity) than the deployed model, rather, it indicates that the extracted model can sometimes generalize better on the test distribution. Fourth, Precision, recall, and F1-score closely mirror the trends observed in accuracy, indicating that the improvements introduced by KD-based MEA are consistent across evaluation dimensions. Interestingly, in several cases, the extracted model slightly outperforms the original model on ground-truth test labels. This behavior aligns with the regularization effects commonly observed in teacher-student distillation frameworks. Crucially, this does not imply higher agreement to the deployed model; rather, it indicates that the extracted model can sometimes generalize better on the test distribution.

### 6.4 Statistical significance

Table 4 reports the *p*-values quantifying the statistical significance of the performance differences between the KD-based MEA and Direct approaches in CF-based evaluation scenarios. This analysis provides a formal test of whether observed discrepancies exceed the variability induced by the pseudo-random components of the methodology and the agreement metric. Results show that without privacy constraints, the KD-based approach exhibits consistently significant improvements over Direct across all datasets and query budgets. Under DP, however, the pattern becomes dataset-dependent: significance persists for Credit, re-emerges for GMSC at higher query budgets, and diminishes for Housing. These results indicate that privacy noise attenuates statistical significance but does not eliminate the underlying performance advantage of KD-based MEA. Overall, the findings suggest that (i) DP introduces additional variance that weakens the detectability of the performance gap, (ii) larger query budgets help recover significance, and (iii) the KD-based approach remains more resilient to DP-induced noise than Direct. In short, DP reduces but does not erase the measurable benefits of KD-based MEA, and its relative robustness becomes increasingly evident as more queries are available.

**Table 4:** *P*-values computed the Mann-Whitney U test.

# Queries	Private	Housing	Credit	GMSC
200	×	0.034	0.019	0.010
1000	×	0.011	0.020	0.010
200	✓	0.824	0.011	0.730
1000	✓	0.188	0.012	0.028

For KD vs. direct under different numbers of queries and privacy settings across datasets.

## 7 Limitations and future directions

We discuss the contribution, limitations of our current approach and potential future research directions:

- Attack Scope: This work is limited to MEA that exploit CFs. Other types of privacy attacks, such as MIA and inversion, are not examined in this paper and therefore fall outside the scope of our current analysis. A future direction is to evaluate whether the proposed mitigation strategy remains effective against this broader class of privacy attacks.
- Privacy-Performance Trade-off: Integrating DP into the CF generator mitigates MEA, but it also introduces limitations by degrading explanation quality, including proximity, prediction gain, and plausibility in some cases. As future work, a more systematic study of the privacy-utility trade-off is needed, including evaluating a wider range of privacy budgets and optimization settings for the DP-based CF generator to better balance protection and explanation quality.
- Focus on Deep Learning Applications: This work focuses on MEA for DNNs, where knowledge distillation is particularly effective due to the expressive capacity of DNNs and their ability to learn rich data representations. This focus limits the applicability of our findings to other model families, such as traditional baselines including tree-based or ensemble methods. Since KD proved effective in the DNN setting, a natural future direction is to explore how KD-based extraction strategies can be adapted to non-DNN algorithms and whether similar performance gains can be achieved.

## 8 Conclusion

In this work, we investigated how counterfactual explanations (CFs) can be leveraged to conduct model extraction attacks (MEAs) via knowledge distillation (KD), and how differential privacy (DP) can be integrated into the CF generation process to mitigate such attacks. Our results show that CFs provide meaningful insights into the decision boundary of the target model, enabling an attacker to train a substitute model even without prior knowledge of the underlying data distribution. At the same time, we demonstrated that incorporating DP into the CF generator can substantially reduce the vulnerability of MLaaS systems by degrading the quality of the extracted CFs and limiting the attacker's ability to reconstruct the target model. Experimental results show that exploiting CFs makes MEA easier, not only increasing their success in terms of agreement but also substantially reducing the number of queries needed to conduct the MEA. Moreover, results show that our proposed KD-based MEA approach outperforms the baseline approach that directly trains the model on the CFs. Beyond demonstrating the feasibility of the proposed MEA and the effectiveness of the DP-based mitigation, our findings highlight several practical implications. First, they underscore that explanation mechanisms, particularly CF generators, should be treated as potentially sensitive outputs, as they may inadvertently reveal structural information about the model. Second, the results emphasize the importance of carefully calibrating privacy mechanisms: while DP protects the training data, it also affects the interpretability and actionability of the generated CFs, creating a privacy-utility tension that MLaaS providers must navigate. Third, our analysis suggests that the security and privacy of explanation-enabled services cannot be evaluated solely in terms of prediction accuracy or explanation fidelity, instead, robustness against extraction must be considered as a core design requirement. Overall, this work provides a foundation for understanding how CF explanations can be leveraged in MEA scenarios and for integrating privacy-preserving mechanisms into CF generation pipelines. The insights gained here open several avenues for future research, including extending the analysis to additional explanation modalities, exploring adaptive DP mechanisms that preserve CF quality, and evaluating the proposed approach across broader model families and data modalities.

## Data Availability

The original contributions presented in the study are included in the article/supplementary material, further inquiries can be directed to the corresponding author.

## Footnotes

1
https://github.com/FatimaEzzedinee/Knowledge-Distillation-Based-Model-Extraction-Attack-using-GAN-based-Private-CFs

2 Experiments were computed with a machine of intel Core i7, a GPU of RTX 3070, and 8 GB of RAM.

3 Note that this analysis is relative to the quality of the generated CFs, and not to the methodology adopted to perform MEA.

4 The focus here is solely on comparing the quality of the generated CFs with and without the integration of DP. This analysis is independent of the specific MEA approach employed and regardless of the number if queries.
