# Correction: Exploiting explanations for model extraction via knowledge distillation and mitigation with private counterfactuals

**DOI:** 10.3389/frai.2026.1925121

**Published:** 2026-08-06

**Authors:** Fatima Ezzeddine, Silvia Giordano, Omran Ayoub

**Affiliations:** 1 Università della Svizzera italiana, Lugano, Switzerland; 2 Scuola universitaria professionale della Svizzera italiana, Lugano, Switzerland

**Keywords:** differential privacy, explainable AI, knowledge distillation, model extraction attacks, responsible AI deployment

There was a mistake in Figure 4 as published. The legend does not accurately reflect the line colors used in the plot.

The corrected Figure 4 legend appears below.

**Figure 4:**
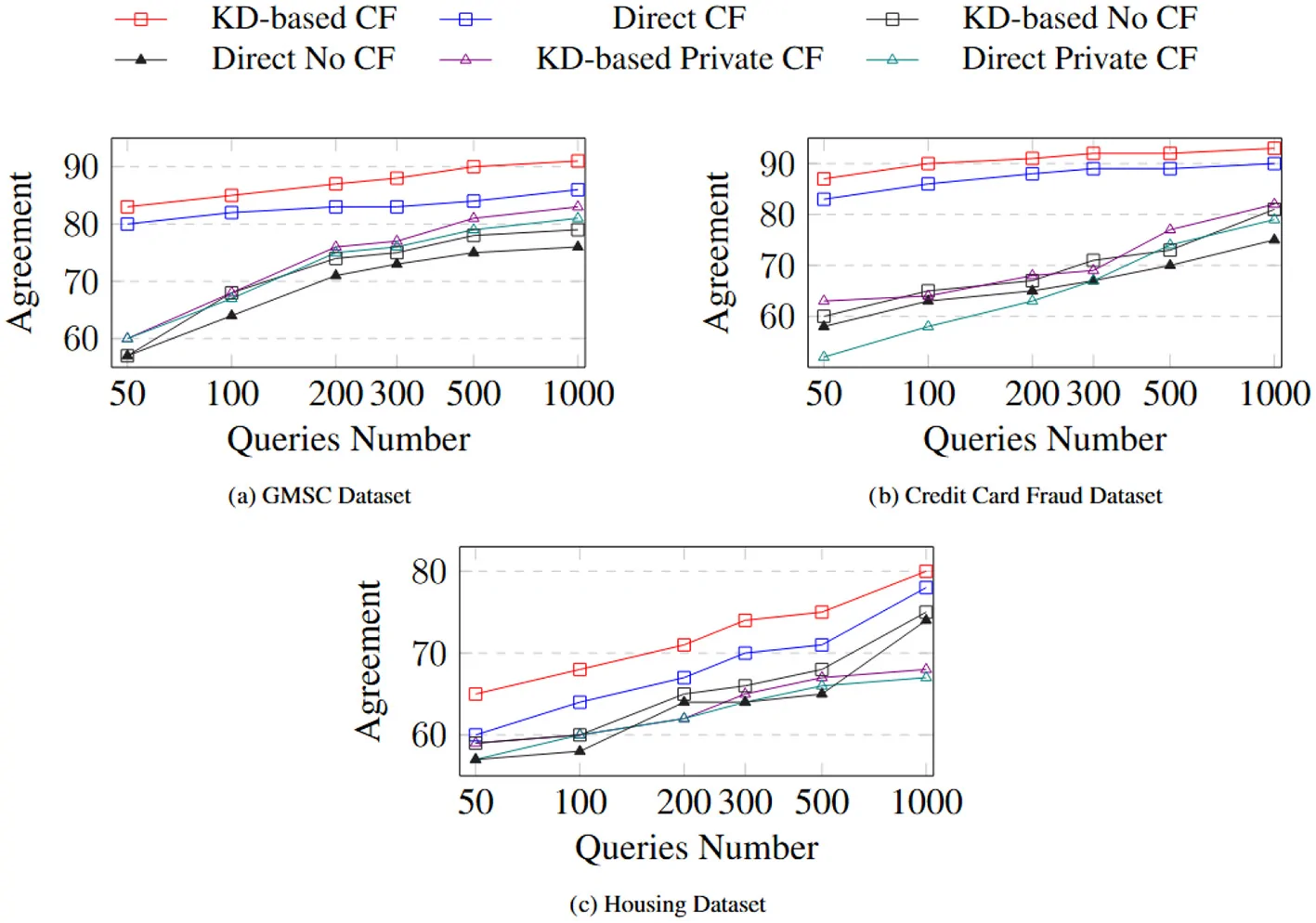
The agreement values achieved by the MEA approach in the various scenarios across the **(a)** GMSC dataset, **(b)** Credit Fraud Dataset, **(c)** Housing Dataset with respect to number of queries made.

The original version of this article has been updated.

